# Supplementary material for: Shared Treg dysregulation underlies psoriasis vulgaris and atopic dermatitis through the PHF19-PRC2 epigenetic axis
Source: Front Immunol. 2026 Aug 20;17:1932788. doi: 10.3389/fimmu.2026.1932788 (PMC13538097; doi:10.3389/fimmu.2026.1932788)
Supplement: Supplementary file 3 [file DataSheet3.zip › Additional files/Additional file 2/12.FAM118A.forest.pdf]

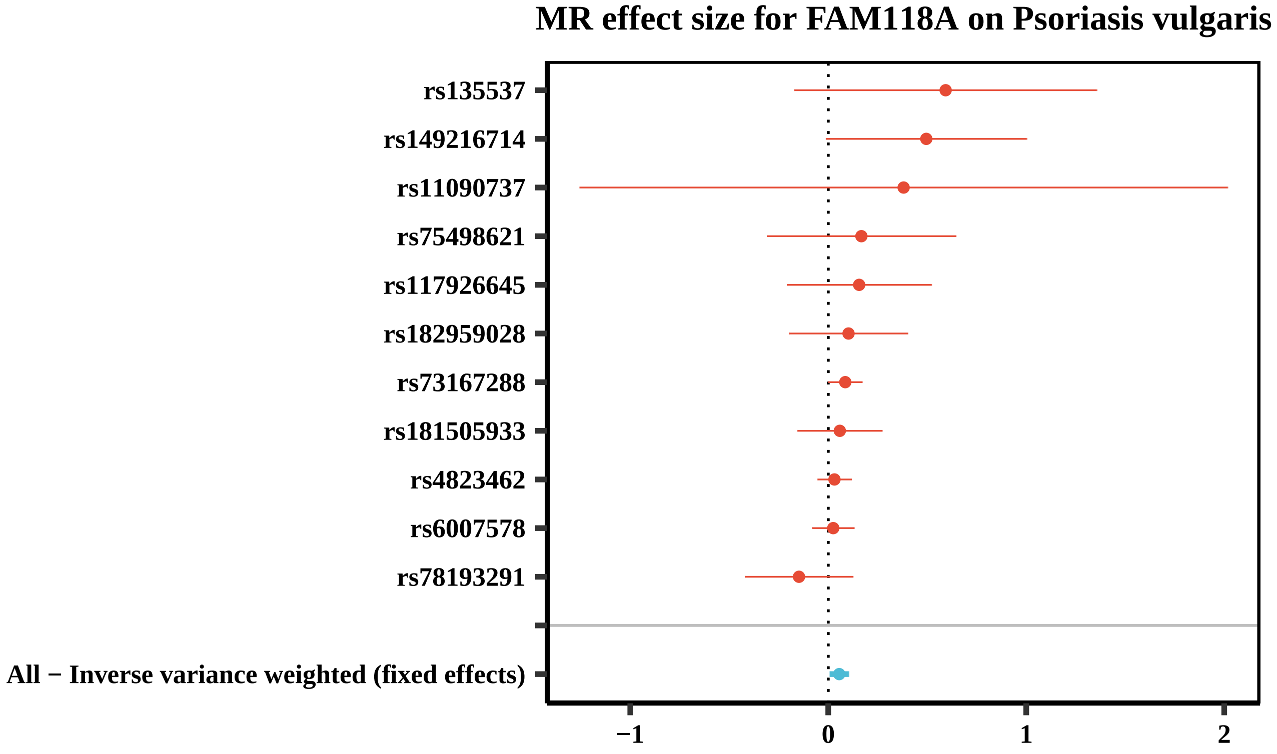
MR effect size for FAM118A on Psoriasis vulgaris
rs135537
rs149216714
rs11090737
rs75498621
rs117926645
rs182959028
rs73167288
rs181505933
rs4823462
rs6007578
rs78193291
All − Inverse variance weighted (fixed effects)
−1
0
1
2
